# Kidney Function, Kidney Function Decline, and the Risk of Dementia in Older Adults: A Registry-Based Study

**DOI:** 10.1212/WNL.0000000000012113

**Published:** 2021-06-15

**Authors:** Hong Xu, Sara Garcia-Ptacek, Marco Trevisan, Marie Evans, Bengt Lindholm, Maria Eriksdotter, Juan Jesus Carrero

**Affiliations:** From the Division of Clinical Geriatrics (H.X., S.G.-P., M.E.), Department of Neurobiology, Care Sciences and Society, Department of Medical Epidemiology and Biostatistics (H.X., M.T., J.J.C.), and Division of Renal Medicine and Baxter Novum (M.E., B.L.), Department of Clinical Science, Intervention and Technology, Karolinska Institutet; Department of Internal Medicine (S.G.-P.), Neurology Section, Södersjukhuset; and Theme Aging (S.G.-P., M.E.), Karolinska University Hospital, Stockholm, Sweden.

## Abstract

**Objective:**

Community-based reports regarding the association between the estimated glomerular filtration rate (eGFR) and dementia risk show conflicting results. The aim of this study is to investigate the links among kidney function, kidney function decline, and dementia incidence.

**Methods:**

We analyzed the association of eGFR with the risk of dementia (defined as a new dementia diagnosis or initiation of dementia treatments) among 329,822 residents of Stockholm who accessed health care during 2006 to 2011, were ≥65 years of age, had no history of dementia, or underwent kidney replacement therapy. We also estimated the rate of eGFR decline among 205,622 residents with repeated eGFR measurements during the first year of observation and investigated its association with subsequent dementia risk.

**Results:**

We detected 18,983 cases of dementia (5.8% of participants) over a median follow-up of 5 years. Dementia incidence rates were progressively higher with lower eGFR: from 6.56/1,000 person-years in those with eGFR of 90 to 104 mL/min to 30.28/1,000 person-years in those with eGFR <30 mL/min. After multivariable adjustment, lower eGFR was associated with a higher dementia risk (hazard ratio [HR] 1.71, 95% confidence interval [CI] 1.54–1.91 in eGFR 30–59 mL/min; HR 2.62, 95% CI 1.91–3.58 in eGFR <30 mL/min) compared with eGFR of 90 to 104 mL/min. A steeper decline in eGFR (decline >2 mL/min/1.73 m^2^/y) within 1 year was associated with higher dementia risk. Risk magnitudes were stronger for vascular dementia than for Alzheimer dementia. As many as 10% (95% CI 6%–14%) of dementia cases could be attributed to eGFR <60 mL/min/1.73 m^2^, a proportion higher than that attributed to other dementia risk factors such as cardiovascular disease and diabetes.

**Conclusions:**

Both lower kidney function and steeper kidney function decline are associated with the development of dementia.

Dementia, the progressive decline of cognition and functioning beyond that of the normal aging process,^1^ occurs mostly in old age, with a prevalence estimate of 5% at 65 to 74 years, 20% at 75 to 84 years, and 50% in those >85 years.^2^ Dementia is associated with increased morbidity^3^ and mortality,^4^ but there are limited treatment strategies.^5^ Currently, identifying potentially modifiable risk factors is the only viable strategy to prevent dementia.^5^

Chronic kidney disease (CKD), the persistent reduction in kidney function, is also very common among older adults, with a population prevalence of 25% to 40% depending on age strata.^6^ Even a mild reduction in kidney function, as defined by estimated glomerular filtration rate (eGFR), is associated with a markedly increased risk of comorbid conditions such as cardiovascular and cerebrovascular disease,^7,8^ infections,^9^ anemia,^10^ and possibly dementia. There is indeed growing evidence of a relationship between the kidneys and the brain.^11-13^ Some studies have shown an association among CKD, a higher prevalence of cognitive impairment, and faster decline in cognitive function.^14-18^ However, previous studies on the association between kidney function and the incidence of dementia diagnosis show conflicting results^19-29^ that might be due to fundamental differences in, for example, methods to assess kidney function or dementia, study population (size and selection criteria), and inclusion of confounders.^26^

Against this background, we decided to explore the risk of dementia across the full spectrum of kidney function in a large population of Swedes ≥65 years of age with serial measures of kidney function.

## Methods

### Study Population

We used data from the Stockholm Creatinine Measurements [SCREAM] project, a health care use cohort from the region of Stockholm, Sweden, including all residents undertaking serum creatinine tests during 2006–2011. Given the commonness of creatinine testing among the elderly, SCREAM captured >90% of the complete population census ≥65 years of age in the region.^30^ Laboratory data were linked with regional and national administrative databases for complete information on health care use, dispensed drugs, validated kidney replacement therapy endpoints (dialysis or transplant), and follow-up for death, with virtually no loss to follow-up. For this study, we included all participants with age ≥65 years at their first available outpatient creatinine measurement, which was considered the study baseline. Exclusion criteria were any recorded history of dementia, missing information on age or sex, and undergoing kidney replacement therapy (maintenance dialysis or kidney transplantation) at cohort entry (figure e-1, doi.org/10.5061/dryad.mw6m905wb).

### Standard Protocol Approvals, Registrations, and Patient Consents

The regional Ethics Committee in Stockholm, Sweden, approved the study protocol. Patient informed consent was not deemed necessary by the ethics committee. Data were deidentified by Swedish government authorities before delivery to the research team.

### Exposures

We first extracted information on all eligible creatinine measurements per individual. Eligible creatinine tests were those performed in an outpatient setting and with plausible concentrations within the range of 0.5 to 17.0 mg/dL. All measurements were standardized to isotope dilution mass spectrometry standards.

The first exposure was eGFR at cohort entry (baseline), which was calculated from the first eligible creatinine for each patient with the Chronic Kidney Disease Epidemiology Collaboration creatinine equation.^31^ Data on ethnicity are not available in Sweden by law, and all participants were assumed to be White. We used 5 eGFR categories per 15–mL/min/1.73 m^2^ increments using the 90 to 104 mL/min/1.73 m^2^ as the reference group. This is consistent with previous work showing that this is an ideal reference because it allows risk to be assessed at higher and lower eGFR.^32,33^

The second exposure was the rate (slope) of kidney function decline (in milliliters per minute per year), calculated from all subsequent eGFR measurements taken within 1 year (±6 months) from the baseline measurement. We set a decrease in eGFR of −1 mL/min/1.73 m^2^ per year as the reference category because this is the expected eGFR change with the normal aging process and may not be pathologic.

### Covariates

Covariates were defined at baseline and included age, sex, and selected comorbid conditions and medications. Definition of comorbid conditions was based on ICD-10 codes. Diabetes, hypertension, and depression were further enriched with information on recent dispensation of related medications. Definitions of medications were based on Anatomical Therapeutic Chemical codes (table e-1, doi.org/10.5061/dryad.mw6m905wb).

### Follow-up and Study Outcome

The primary study outcome was the first recorded diagnosis of all-cause dementia or the initiation of antidementia drugs (donepezil, rivastigmine, galantamine, memantine). The secondary study outcomes were the first recorded diagnosis of Alzheimer dementia and vascular dementia. These and other definitions are detailed in table e-2 (doi.org/10.5061/dryad.mw6m905wb), but we recognize that the validity of these diagnoses may be low. Participants were censored at the end of follow-up (December 31, 2012), death, or migration from the region, whichever occurred first. Death date was obtained from the National Board of Health and Welfare's Cause of Death register (socialstyrelsen.se). The rest of the information was obtained from complete health care records in the region or dispensation at Swedish pharmacies, with presumably no loss to follow-up.

### Data Analysis

Continuous variables were reported as mean ± SD and categorical variables as counts and percentages. Baseline characteristics were compared across different eGFR categories by analysis of variance for continuous variables and Pearson χ^2^ for percentages.

The incidence of all-cause dementia was assessed by the Kaplan-Meier method, and we also calculated incidence rates with 95% confidence intervals (CIs) using the exact method. The proportional hazards assumption was checked with the Schoenfeld residuals test. In the case of violation of the proportionality assumption for covariates, flexible parametric survival models (Royston-Parmar models) were performed to study the association between eGFR and dementia, with attained (chronologic) age as the underlying timescale to better capture the impact of old age on both exposure and outcome. Stepwise adjustments were performed for (1) sex, unhealthy life style diagnoses, and comorbid conditions (2) and medications. We included medications separately from diagnoses because we believe that adds confounding. For example, medications may have various indications (e.g., angiotensin-converting enzyme inhibitor/angiotensin receptor blockers can be used to treat hypertension but also to manage albuminuria in kidney disease) and may add information on the severity of the comorbidity (e.g., hypertension managed with 3 medications is worse than hypertension managed with only 1 medication). We also investigated associations between eGFR and dementia risk using piecewise linear splines, with knots fixed every 15 mL/min/1.73 m^2^ of eGFR.

The weighted contributions of CKD (eGFR <60 mL/min/1.73 m^2^) and other comorbid conditions to the risk of incident dementia were quantified with the population attributable fraction (PAF) with the following equation: PAF = 
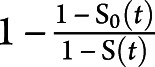
, where S_0_(t) denotes the counterfactual survival function for the dementia event if the exposure would have been eliminated from the population at baseline and S(t) denotes the factual survival function.^34^ The estimates of PAF were adjusted for the abovementioned covariates.

A number of sensitivity analyses were performed to test the robustness of results. First, to explore the possibility of detection bias and reverse causality, we excluded dementia events within the first 2 years of follow-up, in recognition of previous work showing that this is the average time difference between dementia onset and first registry of dementia code.^35^ Second, we explored the association between eGFR categories and all-cause dementia risk in prespecified strata, including sex, age, and presence (versus absence) of selected comorbid conditions.

For those participants who had >1 outpatient eGFR measurement taken within the first year of data collection, we estimated their rate of kidney function decline (eGFR slope) using mixed-effects repeated-measures models of unstructured variance-covariance matrix, random intercept, and random slope. The models were adjusted for baseline eGFR, age, sex, baseline comorbid conditions, and medications. As a next step, we used flexible parametric survival models to relate the eGFR slope to the subsequent dementia risk, and attained age was again set as the underlying timescale. After the exclusion of participants developing dementia during the first year, time at risk began on the date of the last creatinine used to estimate the eGFR slope (new baseline). eGFR slope was introduced as a linear spline with knots at −3, −1, 0, and 1 mL/min/y. The model further adjusted for sex, new baseline eGFR, comorbid conditions, and medications. There were no missing variables to report, and analyses were performed with R (r-project.org, Vienna, Austria) and Stata version 16.0 (StataCorp, College Station, TX).

### Data Availability

SCREAM study data are stored at the Department of Medical Epidemiology and Biostatistics at Karolinska Institutet (ki.se/meb) and can be made available to interested researchers for collaborative projects on request.

Supplementary data are available from Dryad (tables e-1–e-9, figure e-1–e-3): doi.org/10.5061/dryad.mw6m905wb.

## Results

### Baseline Characteristics

There were ≈1.3 million Stockholm citizens accessing health care and undergoing creatinine testing between 2006 and 2011. After exclusion criteria were applied (figure e-1, doi.org/10.5061/dryad.mw6m905wb), the study cohort consisted of 329,822 participants free from dementia history and ≥65 years of age. Their mean age was 74 ± 8 years, and 56% were women (table 1). Mean eGFR was 75 ± 17 mL/min; 0.6% of participants had eGFR ≥105 mL/min, 20% had eGFR of 90 to 104 mL/min , 61% had eGFR of 60 to 89 mL/min , 17% had eGFR of 30 to 59 mL/min , and 1.5% had eGFR <30 mL/min. The most common comorbidity was hypertension (40%), followed by cancer (17%), diabetes mellitus (12%), depression (12%), atrial fibrillation (11%), congestive heart failure (9%), stroke (7%), and myocardial infarction (7%) (table 1).

**Table 1:**
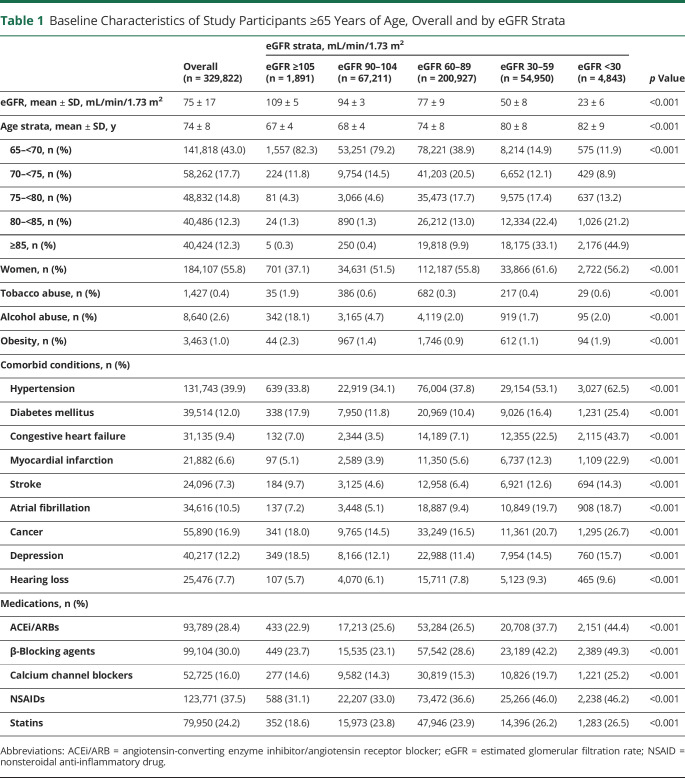
Baseline Characteristics of Study Participants ≥65 Years of Age, Overall and by eGFR Strata

### Incidence Rate and Hazard Ratios of Dementia Across eGFR Strata

Over a median follow-up of 5 years, 18,983 cases of dementia (affecting 5.8% of the participants) were detected throughout 1,185,304 person-years (py). The overall incidence rate was 16.0/1,000 py. Higher incident rates were noted for participants with CKD stage 3 or greater (6.56 per 1,000 py in eGFR 90–104 mL/min [reference group], 15.74 per 1,000 py in eGFR 60–89 mL/min, 26.8 per 1,000 py in eGFR 30–59 mL/min, and 30.3 per 1,000 py in eGFR <30 mL/min separately). The most common dementia diagnosis type was Alzheimer dementia (incident rate 6.36/1,000 py, 2.3% of all participants and 40% of all-cause dementia events), followed by vascular dementia (3.12/1000 py, 1.1% of all participants and 20% of all-cause dementia events). In general, the incidence rate of Alzheimer dementia and vascular dementia increased across lower eGFR categories, except in the category eGFR <30 mL/min for Alzheimer dementia (s-figure e-2 and s-table e-3, doi.org/10.5061/dryad.mw6m905wb). Kaplan-Meier curves suggested higher all-cause dementia risk in participants with lower eGFR categories (s-figure e-3).

**Figure 1:**
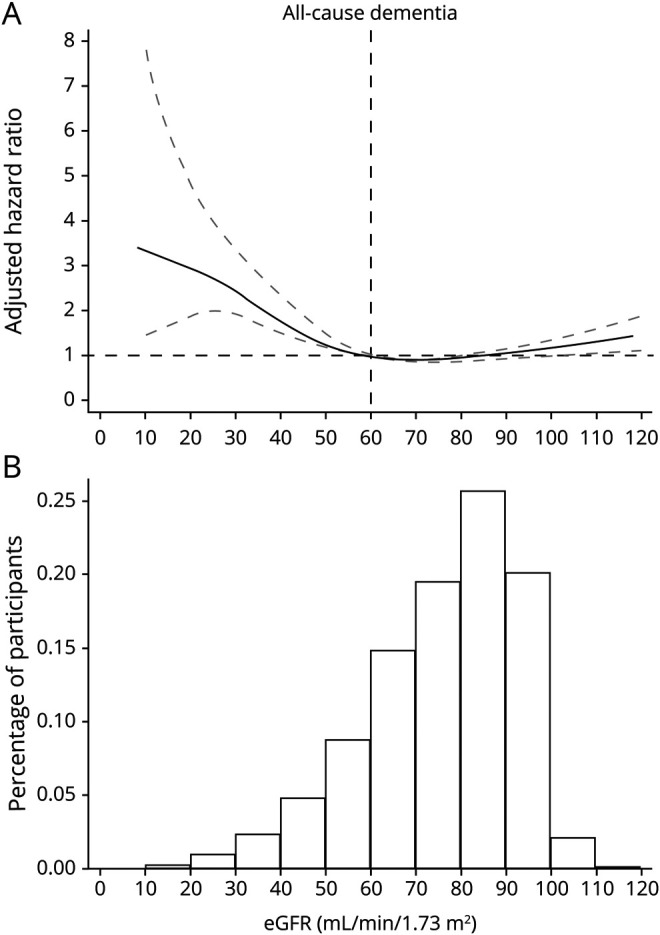
Risk of Dementia Associated With Baseline eGFR (A) Hazard ratios (HRs) for all-cause dementia risk by estimated glomerular filtration rate (eGFR; per 1–ml/min/1.73 m^2^ increase, continuous variable) using linear splines. Model adjusted for sex, chronic smoking, alcohol abuse, diagnosed obesity, comorbid conditions (hypertension, diabetes, congestive heart failure, myocardial infarction, stroke, atrial fibrillation, cancer history, depression, and hearing loss), and medications (use of angiotensin-converting enzyme inhibitor/angiotensin receptor blockers, β-blocking agents, calcium channel blockers, nonsteroidal anti-inflammatory drugs, and statins). Knots were set every 15 mL/min/1.73 m^2^ of eGFR, rendering the same eGFR categories as in table 1. Data were reported as HRs and 95% confidence intervals (dashed lines). (B) Percentage of participants (n = 329,822) across eGFR levels.

Next, we studied whether the higher incidence rates across eGFR strata were explained by accompanying comorbid conditions and characteristics through multivariable-adjusted survival models, selecting eGFR of 90 to 104 mL/min as the reference category. A J-shaped association was observed between eGFR and the risk of all-cause dementia, both when eGFR categories were examined (table 2) and when eGFR was modeled as a continuous metric using splines (figure 1). Compared to participants with eGFR of 90 to 104 mL/min, participants with eGFR of 60 to 89 mL/min had a slightly higher dementia risk (1.16 [1.08–1.26], *p* < 0.001), and those with eGFR of 30-59 and <30 mL/min had 71% (1.71 [1.54–1.91], *p* < 0.001) and 162% (2.62 [1.91–3.58], *p* < 0.001) higher risk of incident all-cause dementia, respectively (table 2).

**Table 2:**
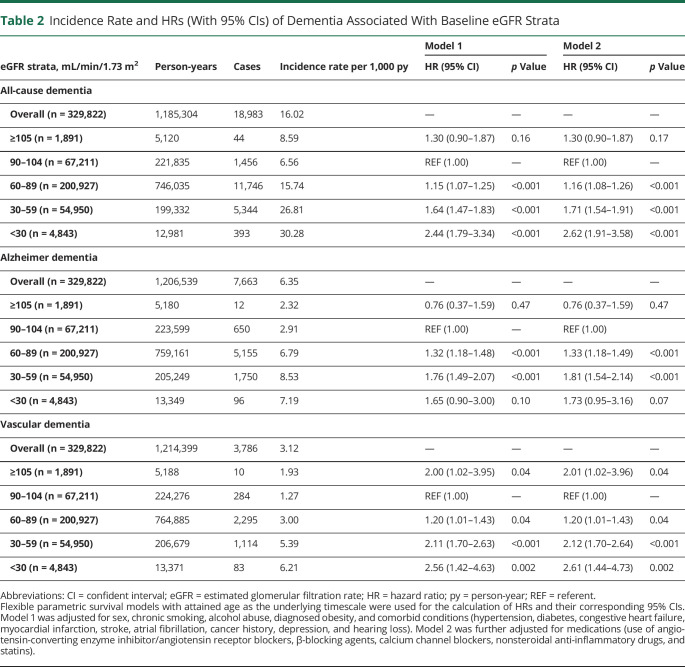
Incidence Rate and HRs (With 95% CIs) of Dementia Associated With Baseline eGFR Strata

Hazard ratios (HRs) for Alzheimer dementia and vascular dementia are shown in table 2. Across lower eGFR categories, we observed a gradually higher risk of Alzheimer dementia and vascular dementia (2.12 [1.70–2.64], *p* < 0.001 in eGFR 30–59 mL/min and 2.61 [1.44–4.73], *p* = 0.002 in eGFR <30 mL/min for vascular dementia; 1.81 [1.54–2.14], *p* < 0.001 in eGFR 30–59 mL/min and 1.73 [0.95–3.16], *p* = 0.07 in eGFR <30 mL/min for Alzheimer dementia). Compared to participants with eGFR of 90 to 104 mL/min, those with eGFR≥105 mL/min had a higher risk of vascular dementia (2.01 [1.02–3.96], *p* = 0.04) but not Alzheimer dementia.

### Plausible Risks Factors and PAF

People who developed dementia had a higher prevalence of comorbid conditions at baseline (table 3). Plausible comorbid risk factors for dementia were explored in multivariable analysis. The HR of dementia risk was highest for the presence of CKD (eGFR <60 mL/min; HR 1.49 [1.23–1.80] *p* < 0.001), followed by having a history of depression (1.49 [1.38–1.62], *p* < 0.001), stroke (1.41 [1.35–1.48], *p* < 0.001), diabetes (1.22 [1.16–1.27], *p* < 0.001), atrial fibrillation (1.10 [1.05–1.15], *p* < 0.001), and myocardial infarction (1.08 [1.02–1.14], *p* = 0.01). The presence of diagnosed hypertension and a history of cancer (0.95 [0.92–0.99], *p* = 0.01; and 0.87 [0.84–0.90], *p* < 0.001 respectively) were associated with a lower dementia risk, and there was no significant association between congestive heart failure and the risk of dementia in our cohort. The use of renin-angiotensin system inhibitors, β-blocking agents, calcium channel blockers, and statins at baseline also was associated with a lower HR of dementia and negative PAFs (table e-4, doi.org/10.5061/dryad.mw6m905wb).

**Table 3:**
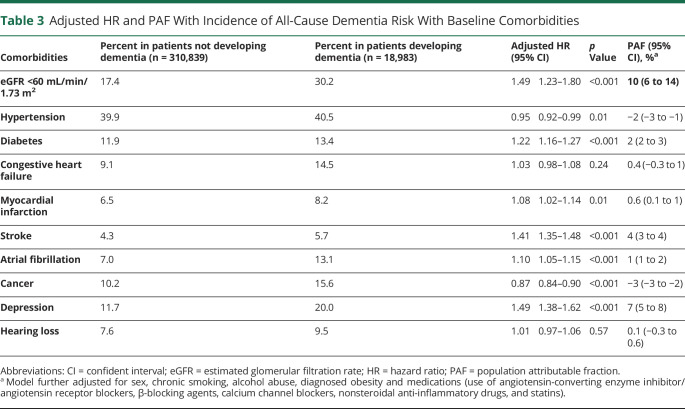
Adjusted HR and PAF With Incidence of All-Cause Dementia Risk With Baseline Comorbidities

Via PAF analyses we estimated that 10% (95% CI 6%–14%) of the dementia cases could be attributed to eGFR <60 mL/min, followed by depression (7% of cases attributed, 95% CI 5%–8%), stroke (4% of cases, 95% CI 3%–4%) and diabetes (2% of cases, 95% CI 2%–3%). PAFs were ≈1% for comorbid conditions such as atrial fibrillation and myocardial infarction (table 3).

### Sensitivity and Subgroup Analyses

We explored potential reverse causation and detection biases by excluding events within the first 2 years of follow-up and still observed associations similar to our main analysis for the lower eGFR strata. Conversely, no association was observed for those with eGFR ≥105 mL/min for all-cause dementia (table e-5, doi.org/10.5061/dryad.mw6m905wb). Stratified analyses are shown in s-tables e-6 and e-7 (doi.org/10.5061/dryad.mw6m905wb). Among all prespecified subgroups, there was suggestion of heterogeneity with lack of association between eGFR and dementia risk in patients ≥75 years of age.

### Associations Between the Rate of eGFR Decline and the Risk of Dementia

There were 205,622 participants (62% of the initial population) who had at least 2 eGFR measurements within the first year of data collection. These individuals were included in the analysis of eGFR change in regard to dementia risk. The median number of plasma creatinine measurements per individual used to calculate the eGFR slope was 3 (interquartile range 2–4), ranging from 2 to 17. During a mean subsequent follow-up of 4.5 years, there were 11,175 cases of all-cause dementia, 4,692 cases of Alzheimer dementia, and 2,495 cases of vascular dementia. The mean eGFR slope was −1.17 mL/min/y (95% CI −1.29 to −1.05, *p* = 0.01) (table e-8, doi.org/10.5061/dryad.mw6m905wb).

**Figure 2:**
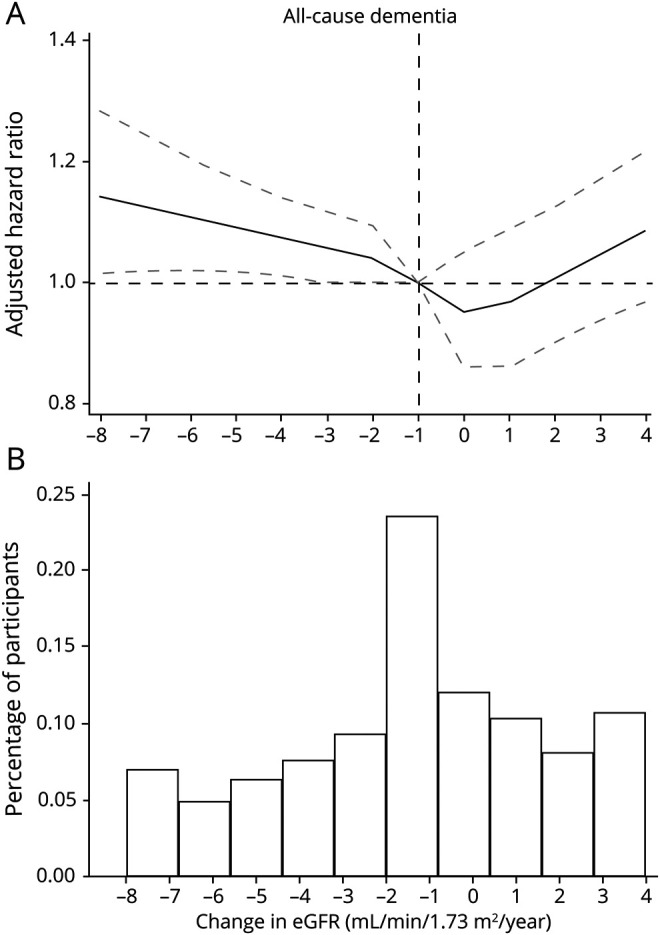
Risk of Dementia Associated With Rate of Kidney Function Decline (A) Hazard ratios (HRs) for all-cause dementia risk by the rate of kidney function decline (estimated glomerular filtration rate [eGFR] slope per ml/min/1.73 m^2^, continuous variable) using linear splines. Model adjusted for baseline eGFR, sex, chronic smoking, alcohol abuse, diagnosed obesity, comorbid conditions (hypertension, diabetes, congestive heart failure, myocardial infarction, stroke, atrial fibrillation, cancer history, depression, and hearing loss), and medications (use of angiotensin-converting enzyme inhibitor/angiotensin receptor blockers, β-blocking agents, calcium channel blockers, nonsteroidal anti-inflammatory drugs, and statins). Knots were set at −3, −1, 0, and 1 mL/min/y. Data were reported as HRs and 95% confidence intervals (dashed lines). (B) Percentage of participants (n = 205,622) across eGFR slope values.

In multivariable-adjusted analyses, a steeper eGFR decline was associated with a higher risk of all-cause dementia (figure 2), with statistically significant associations for declines >2 mL/min/y (table e-9, doi.org/10.5061/dryad.mw6m905wb). Similar associations, but stronger in magnitude, were also observed between the rate of eGFR decline and the risk of vascular dementia. Less clear association was observed for Alzheimer dementia (table e-9, doi.org/10.5061/dryad.mw6m905wb).

## Discussion

This study identifies CKD as a possible risk factor for dementia. In a large register-based cohort of older adults, both lower kidney function and steeper kidney function decline were consistently associated with the risk of dementia diagnosis, particularly vascular dementia. Assuming a direct association, our analysis suggests that 10% of the dementia cases could be attributed to CKD, a proportion higher than that observed for other well-established dementia risk factors such as cardiovascular disease and diabetes.

Our principal finding is an inverse linear association between baseline kidney function and dementia incidence in >325,000 participants. This is, to the best of our knowledge, the largest study to date on the matter, exceeding by several-fold the sample size of all previous studies combined and evaluating the whole spectrum of kidney function. Such results are not novel but may help reconcile the disagreement in previous literature potentially attributed to small sample sizes and dichotomization of kidney function. Our study agrees with and expands on several previous population-based longitudinal studies. First, 2 Japanese studies with collectively 830 older participants reported an increased dementia risk during a mean follow-up of 7 years in individuals with baseline eGFR <60 mL/min.^20,21^ A more recent Japanese study (n = 1,562 participants ≥60 years of age) also observed a higher dementia risk in participants with eGFR <60 mL/min.^27^ Furthermore, the Cardiovascular Health Cognition Study, which recruited 3,349 elderly participants, observed a 37% increase in the risk for dementia incidence and elevated serum creatinine at baseline, but not eGFR, during a mean follow-up of 6 years.^19^ Finally, in the Uppsala Longitudinal Study of Adult Men (n = 1,153), the 11-year risk of Alzheimer dementia also increased with lower eGFR.^22^ In our study, the increased risks observed were stronger for vascular than for Alzheimer dementia, an observation that also agrees with 2 of these previous reports^19,27^ but is inconsistent with another^20^ and was not addressed in the remaining studies.^21,22^

Our findings, however, disagree with several previous studies. First, the French population–based Three-City (3C) study included 7,839 older participants undergoing dementia screening and did not show increased dementia risks for persons with eGFR <60 mL/min at baseline (n = 954), although the stratum of eGFR of 45 to 60 mL/min was associated with increased incidence of vascular dementia.^23^ Second, the US Adult Changes in Thought (ACT; n = 2,968) study found no statistical differences across baseline eGFR strata (n = 1,226 with eGFR <60 mL/min) and the risk of dementia.^25^ In the Sydney Memory and Ageing Study (MAS, n = 889), individuals with lower eGFR (n = 296 with eGFR <60 mL/min) had a decreased risk of cognitive decline or dementia incidence; however, individuals with lower eGFR were more often lost to follow-up.^24^ Finally, in the Systolic Blood Pressure Intervention Trial (SPRINT; n = 8,563 hypertensive adults, age ≥50 years), baseline eGFR <60 mL/min and baseline albuminuria were not associated with risk for dementia during median 5.1 years of follow-up; however, there was an increased risk for probable dementia or MCI among participants with baseline eGFR of 45 mL/min.^36^ The discrepancy between these reports and our study might be explained by differences in study design (screening vs health care extraction) and outcome ascertainment (neuropsychologist performance vs ICD diagnoses/drug dispensations). However, we believe that these previous studies included insufficient numbers of patients with low kidney function, limiting their power to find differences in those ranges. The Norwegian population-based Trøndelag Health Study (HUNT; n = 47,840) found no association between eGFR and dementia.^28^ However, it included participants who were on average 49 years old, which could explain the low proportion of cases with CKD and low number of incident dementia cases (n = 668). Nonetheless, the observation that albuminuria, a marker of early kidney damage, consistently predicted dementia risk offers indirect support to our hypothesis.

Lending credibility to our primary observation, we also report an association between the 1-year rate of kidney function decline and the subsequent (all-cause and vascular) dementia risk among >200,000 patients with repeated creatinine assessments in routine care. Several previous studies have also explored the association between categories of eGFR change and dementia. In the SPRINT study (n = 8,563), declining kidney function measured by eGFR >30% was associated with increased risk for probable dementia, independently of the intensity of antihypertensive.^36^ In a subset (n = 2,382) of participants from the 3C study, a steeper in decline eGFR during the first 4 years of follow-up was associated with higher risk for all-cause and vascular dementia but not for Alzheimer dementia.^23^ However, the ACT study (n = 2,968) failed to observe any association between eGFR decline within 6 years and risk of dementia.^25^

We noted the higher end of eGFR (≥105 mL/min) in our population, and the few participants who appeared to have increased their kidney function during the first year were also at increased risk for all-cause and vascular dementia. This should not be interpreted as higher kidney function/kidney function improvement conferring increased risk. We attribute it to the inherent error of eGFR measurements in the context of other underlying diseases. In our data from routine care, higher eGFR measures most likely represent inaccurate estimations of true GFR caused by low serum creatinine, which usually accompanies conditions of reduced muscle mass or chronic illness.^37^ Body weight loss and malnutrition often precede dementia diagnosis,^38,39^ and these conditions may also affect the eGFR estimations.^40^ On the other hand, we cannot exclude the possibility that patients with eGFR ≥105 mL/min may de facto have glomerular hyperfiltration, and we note that the prevalence of diabetes and diagnoses of obesity, alcohol abuse, or chronic smoking (all of which have been suggested to increase the risk of glomerular hyperfiltration and dementia risk) were higher for this category.^41^ This being said, our results agree with a recent Korean study^42^ in which dementia risk, in particular vascular dementia, was higher in persons with eGFR above the 95th percentile of distribution compared with those in the 50th to 65th percentile.

Several plausible mechanisms explain the link between CKD and dementia. First, the shared traditional vascular risk factors of aging, hypertension, diabetes, and hypercholesterolemia lead to cerebrovascular injury, dementia, and kidney disease.^43^ Second, consequences of CKD, including chronic inflammation and oxidative stress,^44^ endothelial abnormalities,^45^ disorders of the immune system,^46^ or hypercoagulation,^43^ are associated with the prevalence and occurrence of ischemic cerebrovascular lesions. Third, kidney dysfunction causes retention of uremic toxins such as cystatin C, guanidine compounds, and hyperhomocysteinemia, which results in direct neurologic toxicity to the cerebral cortex, the mammillary bodies, and the thalamus.^47,48^ This neurologic toxicity would lead to clinical/subclinical disease (silent brain infarcts, microbleeds or stroke, and white matter lesions), which is known to contribute to vascular and nonvascular cognitive impairment and possibly increased dementia risk.^11-13^ Finally, persons with CKD have frequent microvascular and macrovascular complications,^49^ leading to an increased risk of cardiovascular events and stroke,^50^ which may be intermediates in the associations observed.

An important limitation in our study is the reliance on ICD diagnoses of dementia.^51^ Although the capture of these is estimated to be complete for our region, access to medical records would have refined the accuracy of diagnoses and probably allowed us to identify additional cases. Enriching our outcome with data on initiation of dementia medications probably improves this, but there remains a large proportion of older persons with undetected dementia living in the community,^52^ and the risk of misclassification bias exists. Furthermore, we rely on issued diagnoses and did not have access medical records; there may be patients thought to have dementia or predementia by their physicians who lack a diagnosis. Because the distinction of dementia types by clinical diagnoses may not always be accurate, our results concerning these may be considered hypothesis generating. For instance, patients with a history of stroke may have more often been diagnosed as having vascular dementia, and mixed Alzheimer and vascular dementia is probably more common than pure vascular dementia. Collectively, we would have benefited from confirming our diagnoses via linkage with the Swedish Dementia Register, which contains confirmation of dementia diagnoses with Mini-Mental State Examination score and types of dementia as confirmed by the clinician doing the examination.^53^ The testing of eGFR is not standardized, which may lead to detection bias (that is, patients with specific indications may be subjected to more frequent eGFR testing than others, or eGFR testing is performed as part of the workup toward a diagnosis of dementia). Our analyses excluding diagnoses within the first 2 years of follow-up or adjustment for the number of tests in the mixed model tried to evaluate the impact of these biases. Our median follow-up time can be considered short to study the overall incidence of dementia, but a longer follow-up may contribute to misclassification bias, given that kidney function also declines with time. We have no information on lifestyle, body mass index, or muscle mass, but we included clinical diagnoses of alcohol abuse, chronic smoking, and obesity. While we recognize that the sensitivity of these lifestyle-related diagnoses is low, they are highly specific and may adjust for some additional confounding. Finally, as in all observational and register-based analyses, causality cannot be inferred, and we acknowledge the existence of residual and unknown confounding, something we tried to tackle though in our sensitivity analyses.

This large health care study found an increased dementia incidence in individuals with CKD and rapid kidney function decline. Our findings may help health care policy makers to develop and implement appropriate strategies for screening and monitoring for dementia in persons with CKD (and vice versa) and assist in health service planning. On one hand, we speculate that estimating kidney function at dementia screening visits may help in risk stratification, provision of lifestyle recommendations, and perhaps consideration of cholinesterase inhibitors over other antidementia medications, given their potential kidney function–sparing effect.^54^ On the other hand, because awareness of CKD is still very low among patients and physicians,^30^ such testing may allow early identification of CKD cases who can benefit from nephrologist referral and initiation of antiproteinuric therapies (i.e., renin-angiotensin system inhibitors). We note that a recent meta-analysis of prospective cohort studies observes consistency of evidence regarding a lower dementia risk associated with the use of renin-angiotensin system inhibitors in patients with high blood pressure.^55^ Lifestyle habits that contribute to the prevention of cardio-cerebral vascular disease may reduce the risk of dementia and reduce the risk of CKD progression.^7,8,56^

## Glossary

ACT: Adult Changes in Thought
CI: confidence interval
CKD: chronic kidney disease
eGFR: estimated glomerular filtration rate
HR: hazard ratio
HUNT: Trøndelag Health Study
ICD-10: *International Classification of Diseases, 10th revision*
MAS: Sydney Memory and Ageing Study
PAF: population attributable fraction
py: person-years
SCREAM: Stockholm Creatinine Measurements
SPRINT: Systolic Blood Pressure Intervention Trial
3C: Three City
